# Circulating Biomarkers in Sarcoma

**DOI:** 10.1007/s11912-025-01692-0

**Published:** 2025-06-17

**Authors:** Taylor P. Williams, Valerie P. Grignol

**Affiliations:** Division of Surgical Oncology, Department of Surgery, The Ohio State University Wexner Medical Center, 460 W. 10 th Ave, Columbus, OH 43210 USA

**Keywords:** Biomarkers, Sarcoma, CtDNA, Extracellular vesicles

## Abstract

**Purpose of Review:**

Biomarkers to evaluate, diagnose, and monitor cancer treatment can provide critical adjunct information to standard methods of cancer detection and monitoring. With over 100 different histologic subtypes, sarcomas are a group of tumors with a critical need for easily retrievable circulating biomarkers. This review presents the current progress in the field. We describe the myriad types of biomarkers, highlight the modalities of testing, and explore the clinical utility of specific biomarkers.

**Recent Findings:**

Genomic circulating biomarkers include circulating tumor DNA and micro-RNA. Genomic expressions for different histologic subtypes of sarcoma are highlighted in this review. We also explore different oncogenes, their respective proteins, extracellular vesicles, and dysregulated metabolites as investigational circulating biomarkers for diagnostic and prognostic purposes.

**Summary:**

Soft tissue sarcomas are a category of cancers that lack a clinically validated circulating biomarker, and this review presents the ongoing research efforts to establish a genomic, proteomic, or metabolic biomarker.

## Introduction

Soft tissue sarcoma (STS) is an uncommon malignancy comprising 1% of adult solid tumor [1]. There are over 100 histological subtypes of sarcoma, making it difficult to conduct subtype-specific analyses given the small incidence of each subtype. As a result of the rarity and heterogeneity of sarcomas, new therapeutic and diagnostic tools for advanced sarcoma have been slow in their development.

Circulating biomarkers refer to any number of molecules that may be obtained in bodily fluids, such as circulating tumor cells, exosomes, proteins, or nucleic acids [2]. Circulating biomarkers show promise as a possible target for diagnostic testing, which may provide information about disease prognosis and guide treatment strategies [3]. Biomarkers are quantitative and have distinct sensitivities and specificities dependent on the methodology by and purpose for which they were obtained. Established biomarkers in other cancers have utility in screening, diagnosing, and treating early, recurrent, and metastatic disease. The term liquid biopsy has been used to describe several different methodologies by which blood and its components, tears, stool, saliva, cerebrospinal fluid, or urine have been used to obtain specific circulating biomarkers [4]. The liquid biopsy specimen is processed using various assays to identify biomarkers, and, unlike tumor genomic testing, these liquid biopsies may be taken repeatedly at different intervals over the course of the patient’s disease [5].

To date, no circulating biomarker has been clinically validated and established for any purpose in the diagnosis or management of STS. In this review, we will present the current state of investigations of circulating biomarkers in STS. We will focus on genomic, including circulating tumor DNA (ctDNA) and micro-RNA(miRNA), proteomic, and metabolic biomarkers that show promise in the field of sarcoma.

## Genomic Circulating Biomarkers

### Circulating Tumor and Cell-Free DNA

Cell-free DNA(cfDNA) is released by cells, following cell death and lysis, into the peripheral blood where it may be isolated and quantified. When tumor cells experience apoptosis, they release cfDNA which may then be referred to as circulating tumor DNA, or ctDNA [6]. Only a small fraction of a patient’s overall cfDNA will be ctDNA and only a fraction of patients with advanced malignancies will have detectable ctDNA [7].

Several methods are used in the quantification of cfDNA and ctDNA: next generation sequencing (NGS), whole-genome sequencing (WGS), and polymerase chain reaction (PCR). Several custom assays have been developed for NGS, WGS, and PCR [8]. The specificities for detecting ctDNA in known cancer patients have been published and are very high (> 95%) for most assays, such as the CancerSEEK PCR or the CAPPseq NGS panels; however, sensitivities are widely variable by modality and cancer type [9–11]. Roche’s cobas® PCR assay was the first platform to gain approval from the US FDA to evaluate for epidermal growth factor receptor mutations in patients with non-small cell lung cancer [12]. To date, no such platform has been approved for use in sarcoma. CAPPseq had both high sensitivity and specificity in a small group of seven patients with leiomyosarcoma. A larger sample of patients would need to be tested to further validate this method. Other attempts to isolate ctDNA in sarcoma have not been as successful. Eastley et al. used a custom amplicon panel which was only able to isolate ctDNA from one in 40 patients with known sarcoma from a mix of subtypes [13]. They also used Ion Ampliseq™ NGS and a custom-designed PCR assay but were only able to isolate ctDNA in 17% of patients. Moreover, there was no correlation between cfDNA levels and tumor size, grade, recurrence, or histologic subtype, leading the authors to conclude that circulating nucleic acid analysis in sarcoma was not yet clinically useful. Perhaps the most clinically successful case for utilizing ctDNA in the management of sarcoma comes from the published results of the phase III INTRIGUE trial [14]. The trial randomized patients with advanced gastrointestinal stromal tumors who had failed therapy with imatinib to receive either ripretinib or sunitinib with the primary endpoint of progression-free survival (PFS). As part of an exploratory analysis, ctDNA levels were obtained using an NGS assay, with detectable levels in 77% of samples. They were able to sequence the ctDNA to evaluate specific KIT mutations which they found had significantly different PFS depending on the systemic treatment received. This impressive result is the strongest evidence of the use of ctDNA to drive therapy in sarcoma to help oncologists determine the optimal second line therapy for select patients with GIST.

Like the efforts of Eastley et al., as previously described, other studies have been published evaluating the overall quantification of ctDNA and its correlation to different patient outcomes. Braig et al. showed that plasma concentrations of cfDNA levels are higher in STS patients with known disease compared to healthy controls or STS patients in remission, suggesting that cfDNA concentrations alone may be useful in detecting recurrent disease [15]. They then investigated specific mutational breakpoints in different histologic subtypes and found a correlation between the detection of an established mutation in myxoid liposarcoma and the patient’s overall tumor burden. This study suggests that detection of ctDNA may be useful to shorten the interval of visits or diagnostic imaging in surveillance to detect recurrences in a timelier fashion. Runkel et. al. evaluated ctDNA with NGS from blood samples of patients with high-grade STS who received neoadjuvant radiation therapy [16]. They found characteristic patterns in concentrations of ctDNA over the course of neoadjuvant radiation and paired these data with serial MRIs. Their preliminary data suggest that ctDNA levels may correlate with tumor volumes and, as a result, ctDNA may be a promising surrogate for response to therapy. Further investigation is warranted to substantiate this claim. Lak et al. used WGS to isolate cfDNA from plasma samples in 16 of 30 pediatric patients with rhabdomyosarcoma [17]. They were able to correlate the positive detection of the RASSF1 A-M mutation with worse event free survival, making the study a strong starting point for the use of liquid biomarkers in a pediatric population with a rare and aggressive STS.

ctDNA has also been investigated as a biomarker for minimal residual disease, to predict the risk of relapse in patients following curative resection [18]. Minimal residual disease refers to any residual tumor following surgery which may provide the seed for recurrent tumor growth. Although the isolation of ctDNA from minimal residual disease may not be sensitive enough at this time to provide reliable data, it may be feasible in the future, as technology improves, that ctDNA from a small number of residual tumor cells might be detectible.

There are technological limitations in the quantification and sequencing of ctDNA; however, that technology is improving. We have seen the landscape of testing evolve from numerous custom assays into more standardized commercial platforms. Clonal hematopoiesis is the process of accumulating somatic mutations in hematopoietic stem cells with aging, and the detection of these non-tumor mutations during ctDNA analysis of plasma is a source of biological background noise [19]. These confounding mutations continue to provide a technical challenge with this modality of testing. Identification of specific mutations and sequences for each histologic subtype remains a challenge in STS. With increasingly powerful bioinformatics, the identification of relevant sequences detected in ctDNA will only continue to improve.

### Micro-RNA

Many of the early efforts in studying circulating miRNA in sarcoma have focused on their diagnostic and prognostic potential. These miRNAs alter the gene expression of specific target oncogenes and thus influence cell growth and differentiation. The dysregulation of specific miRNAs, be it under expression or over expression, is known to contribute to the development and spread of cancers such as STS [20]. Multiple studies have aimed to catalog these different expression patterns and associate them with specific subtypes of STS [21].

In liposarcoma (LPS), approximately 90% of tumors have an overexpression of the mouse double minute 2 (MDM2) protein, which is associated with the overexpression of miR-26a-2 [22]. The overexpression of miR-26a-2 has also been correlated with poor patient survival in LPS, making it a known circulating biomarker and a possible therapeutic target whose repression may reduce LPS progression [23]. Fricke et al. identified both miR-3613-3p and miR-4668-5p were upregulated in blood samples taken from patients with dedifferentiated LPS when compared to healthy control patient blood [24]. The same group was also able to identify a panel of seven miRNAs that were significantly upregulated in patients with active synovial sarcoma compared to healthy controls and patients in complete remission [25]. These are just a few of the many examples of miRNA targets that have been identified as subtype specific biomarkers; however, their clinical utility has not yet been established.

Asano et al. used serum samples from over 1000 patients with STS to evaluate miRNA profiles for more than 43 histological subtypes of STS [26]. They identified seven miRNA signatures and developed a molecular detector called IndexVI. The sensitivity and specificity of IndexVI was 90% and 95%, respectively, for identifying patients with STS compared to healthy and benign control patients; however, IndevVI was unable to distinguish between different histologic subtypes. Studies like this will provide the foundation for advances in analyzing circulating miRNA for diagnostic purposes in STS.

## Proteomics

There is a paucity of studies on the use of proteomics to identify circulating biomarkers, with most work focusing on the analysis of solid tumor samples. As techniques in both immunohistochemistry and mass spectrometry have improved, so has the promise of proteomic strategies in sarcoma research [27].

Cervi et al. studied the platelet angiogenesis proteome in mice and sought to evaluate platelet-associated platelet factor-4 (PF-4) as a potential cancer biomarker in serum [28]. Using mass spectrometry and immunoassays with anti-PF-4 antibody, they were able to show upregulation of PF-4 in early growth of liposarcoma and osteosarcoma. The study, published in 2008, was an early look at protein biomarkers such as PF-4; however, to our knowledge, this work has not been further investigated.

Programmed cell death ligand 1 (PD-L1) is a protein in the immune checkpoint pathway which regulates innate T-cell response to malignancy [29]. As immunotherapy with immune-checkpoint inhibitors (ICI) have become increasingly used, circulating PD-L1 protein has been studied as a biomarker for response to ICIs. High serum levels of PD-L1, determined by enzyme-linked immunosorbent assay (ELISA), have been associated with poor survival in the setting of systemic therapy with ICIs [30]. A meta-analysis of PD-L1 expression and clinical outcome found that high PD-L1 expression was correlated with poor overall survival and event-free survival in patients with both bone and soft-tissue sarcoma [31]. While these two examples show how proteomics may be used to identify circulating biomarkers in STS, there is still much more work to be done before they are clinically relevant.

## Subtype-specific Biomarkers

There are some biomarkers which have been studied in specific sub-types of STS. MDM2 has been studied as a predictive biomarker in LPS in addition to its use in diagnosing LPS [32, 33]. Extracellular vesicles (EVs), or exosomes, are nanoparticles that contain DNA, RNA, and proteins which aid cell communication within the tumor microenvironment [34]. The contents of these EVs are referred to as cargoes. Because EVs circulate in the plasma, their cargoes may be targeted as circulating biomarkers. Our group isolated and measured MDM2 DNA from EVs using PCR in patients with both well-differentiated (WD) and dedifferentiated (DD) LPS before and after surgical resection and compared levels to those of healthy donors. MDM2 levels were significantly higher in pre-surgical LPS patients in comparison to both healthy donors and post-resection LPS patients. Increases in MDM2 levels post-resection were correlated to recurrent or persistent disease, suggesting that EV isolated MDM2 may be a circulating biomarker for early recurrence in LPS. A study by Bill et.al., also from our institution, supports this conclusion [35]. Using data from three clinical trials, increased MDM2 genomic amplification and mRNA expression in patients with DD LPS following complete tumor resection was associated with shortened recurrence free survival and overall survival.

High-mobility group box 1 (HMGB1) is a protein released from cells as a result of immunogenic cell death and can be isolated in plasma; HMGB1 was recently studied as a potential biomarker in a Phase 1b clinical trial treating patients with advanced leiomyosarcoma (LMS) with doxorubicin, dacarbazine, and an ICI, nivolumab [36, 37]. Elevated levels of HMGB1 correlated to longer PFS in the trial; however, these results are based on a small data set and must be interpreted in the context of the treatment regimen of the trial.

In Ewing sarcoma (ES), circulating tumor cells carry the EWS-FLI1/ERG chimeric transcript, which can be isolated from bone barrow or peripheral blood in 19–43% of patients, and this target has long been thought to be a potential clinical biomarker for ES patients [38, 39]. A study by Przybyl et al. found that EWS-FLI1/ERG in either tumor or peripheral blood samples did not have prognostic value; however, they did find that cadherin 2 (CDH2) downregulation in peripheral blood samples of ES patients correlated to worse overall survival.

Mihaly et.al. were able to detect the chimeric SS18-SSX fusion gene in peripheral blood samples of patients with synovial sarcoma; however, they were only able to do so in one of 15 patients, leading them to conclude that this cell-free nucleic acid was not a useful target as a circulating biomarker [40].

## Dysregulated Metabolites

Some work has been done to evaluate metabolite levels in the blood to determine if there is any prognostic value in STS patients. Miolo et. al. evaluated a panel of over 68 metabolites using liquid chromatography-tandem mass spectrometry of free serum amino acids and found that only citrulline and histidine correlated with overall survival [41]. With that data they devised a risk prediction model using citrulline, hemoglobin, and performance status to classify metastatic STS patients according to expected overall survival. As part of a phase II trial treating STS patients with pembrolizumab in combination with metronomic cyclophosphamide, Toulmonde et. al. discovered that patients in the study had a significant increase in their plasma kynurenin to tryptophan ratio [42]. Kynurenin is a metabolite of tryptophan produced by indoleamine 2,3-dioxygenase 1(IDO1) which promotes T cell expansion and may be a target biomarker for patients receiving ICIs that affect the IDO1 pathway.

## Conclusion

Soft tissue sarcomas are a category of cancers that lack a clinically validated circulating biomarker, and this review presents the ongoing research efforts to establish a genomic, proteomic, or metabolic biomarker. Technological advances have improved the investigation of specific circulating proteins and nucleic acids with the promise that clinically valuable biomarkers may soon be available through liquid biopsy of patients with STS.

## Key References

- Przybyl J, Chabon JJ, Spans L, et al. Combination approach for detecting different types of alterations in circulating tumor DNA in leiomyosarcoma. Clin Cancer Res. 2018 Jun 1;24(11):2688–2699. 10.1158/1078-0432.CCR-17-3704.
  o This study highlights a promising approach to detecting ctDNA in a small group of patients with leiomyosarcoma
- Heinrich MC, Jones RL, George S, et al. Ripretinib versus sunitinib in gastrointestinal stromal tumor: ctDNA biomarker analysis of the phase 3 INTRIGUE trial. Nat Med. 2024 Feb;30(2):498–506. 10.1038/s41591-023-02734-5.
  o This clinical trial provides the strongest evidence supporting the use of ctDNA to determine the optimal second line therapy in patients with GIST
- Runkel A, Braig D, Bogner B, et al. Non-invasive monitoring of neoadjuvant radiation therapy response in soft tissue sarcomas by multiparametric MRI and quantification of circulating tumor DNA—a study protocol. PLoS One. 2023 Nov 1;18(11):e0285580. 10.1371/journal.pone.0285580.
  o This study was the first to evaluate concentrations of ctDNA in patients receiving neoadjuvant radiation for STS
- Lee DH, Amanat S, Goff C, et al. Overexpression of miR-26a-2 in human liposarcoma is correlated with poor patient survival. Oncogenesis. 2013;2(5):e47. 10.1038/oncsis.2013.10.
  o This study shows a significant correlation between miRNA expression and patient survival in liposarcoma
- Asano N, Matsuzaki J, Ichikawa M, et al. A serum microRNA classifier for the diagnosis of sarcomas of various histological subtypes. Nat Commun. 2019;10(1):1299. 10.1038/s41467-019-09143-8.
  o This study is the most comprehensive attempt at cataloging miRNA profiles for each histologic subtype of STS
- Calore F, Casadei L, Sarchet PD, et al. Extracellular vesicle-MDM2-DNA as a potential liquid biopsy biomarker for disease identification in retroperitoneal liposarcoma. Ann Surg. 2024 May 21. 10.1097/SLA.0000000000006345.
  o In this study, quantification of MDM2 isolated from EVs was explored as a biomarker of early recurrent or post-operatively persistent WD/DD retroperitoneal liposarcoma
- Bezu L, Kroemer G. High circulating HMGB1 indicates good prognosis in patients with advanced leiomyosarcoma under chemoimmunotherapy. Oncoimmunology. 2024 Dec 31;13(1):2432059. 10.1080/2162402X.2024.2432059.
  o This study evaluated HMGB1 as a circulating biomarker to gauge treatment response in leiomyosarcoma patients receiving chemoimmunotherapy

## Data Availability

No datasets were generated or analysed during the current study.
